# Novel Insights Into the Immune-Regulatory Functions of Mast Cells in the Cutaneous Immune Response

**DOI:** 10.3389/fimmu.2022.898419

**Published:** 2022-05-12

**Authors:** Tetsuya Honda, Yuki Honda Keith

**Affiliations:** ^1^ Department of Dermatology, Hamamatsu University School of Medicine, Hamamatsu, Japan; ^2^ Department of Dermatology, Kyoto University Graduate School of Medicine, Kyoto, Japan

**Keywords:** mast cell, IL-10, PD-1, regulatory T cells (Tregs), regulatory B cells (Bregs)

## Abstract

Skin is a frontline organ that is continuously exposed to external stimuli, including pathogens. Various immune cells reside in the skin under physiological conditions and protect the body from the entry of pathogens/antigens by interacting with each other and orchestrating diverse cutaneous immune responses. To avoid unnecessary inflammation and tissue damage during the elimination of external pathogens and antigens, skin possesses regulatory systems that fine-tune these immune reactions. Mast cells (MCs) are one of the skin-resident immune cell populations that play both effector and regulatory functions in the cutaneous immune response. So far, the interleukin-10-mediated mechanisms have mostly been investigated as the regulatory mechanisms of MCs. Recent studies have elucidated other regulatory mechanisms of MCs, such as the maintenance of regulatory T/B cells and the programmed cell death protein-1/programmed cell death-ligand 1-mediated inhibitory pathway. These regulatory pathways of MCs have been suggested to play important roles in limiting the excessive inflammation in inflammatory skin diseases, such as contact and atopic dermatitis. The regulatory functions of MCs may also be involved in the escape mechanisms of antitumor responses in skin cancers, such as melanoma. Understanding and controlling the regulatory functions of skin MCs may lead to novel therapeutic strategies for inflammatory skin diseases and skin cancers.

## Introduction

Skin is the outermost organ separating the body from the outer environment. As the skin is constantly exposed to various external stimuli, such as pathogens and physical/chemical trauma, it has sophisticated barrier systems that protect the body from such stimuli. One such barrier system includes the immunological barrier, in which skin-resident or infiltrated immune cells exhibit effector functions to eliminate the pathogens (1). To form this barrier, the immune cells perform effector functions by interacting with each other and producing various cytokines/chemokines, leading to inflammation. While inflammation is necessary for efficient protection of the body from external stimuli such as pathogens and antigens, excess of it can cause undesired tissue damage. To minimize tissue damage, multiple regulatory systems must precisely control the effector functions of immune cells.

Among the immune cells in the skin, mast cells (MCs) are one of the most abundant skin-resident immune cells, accounting for approximately 10% of all hematopoietic cells in the skin (2). Skin MCs consist in connective tissue-type MCs (CTMCs) in mice and tryptase-positive, chymase-positive MC_TC_ in humans and are considered to be derived from the bone marrow, yolk sac, and the aorta-gonad-mesonephros region (3–6). Under physiological conditions, MCs are localized in the hypodermis and dermis, mainly around blood vessels, nerve fibers, and hair follicles (7, 8). Several studies have been performed to elucidate the roles of MCs in the skin under steady and diseased conditions. MCs play both facilitating and regulatory roles in a context-dependent manner (9–13). So far, the interleukin (IL)-10-mediated mechanisms have mostly been investigated as the regulatory mechanisms of MCs. However, recent studies have revealed several IL-10-independent regulatory functions of MCs.

In this review, we discuss the recent findings on the immunoregulatory functions of skin MCs and their underlying mechanisms in the context of inflammatory skin diseases and skin cancers, focusing on contact dermatitis, atopic dermatitis (AD), and malignant melanoma (**Table 1**).

**Table 1:** A summary of references regarding the regulatory functions of mast cells in the skin.

Mouse model	Regulatory mechanisms (reference)
Contact hypersensitivity	Production of IL-10 (14, 15) Maintenance of Bregs via IL-5 (16) Reduction of T_RM_s by degradation of IL-15 (17) PD-1/PD-L1 signaling (18)
Atopic dermatitis	Maintenance of Tregs via IL-2 (19)
Malignant melanoma	Recruitment of Tregs (20) Induction of M2 macrophages by histamine (21)

## Contact Dermatitis 

Contact dermatitis, such as metal allergy and plant allergy, is a prototypic Th1/Type 1 CD8^+^ T cells (Tc1)-type immune response of the skin (1). Mouse contact hypersensitivity (CHS) is one of the most frequently used animal models of contact dermatitis (22). Small molecules (<500 Da), called haptens, bind to self-proteins and become antigens to induce CHS. When foreign antigens invade the skin, they are captured by the skin dendritic cells (DCs), mainly dermal DCs, which subsequently migrate to the skin-draining lymph nodes (LNs) and undergo maturation. The migrated DCs then present the antigens to naive T cells in an antigen-specific manner and promote their differentiation into effector T cells (sensitization). When the same antigens enter the skin, the skin DCs present the antigens to skin-infiltrated effector T cells *in situ* and activate them to produce cytokines and cytotoxic molecules, such as interferon-γ and granzyme B, which lead to antigen-specific immune responses (elicitation). MCs play a role in CHS regulation *via* multiple mechanisms.

### Promotion of CHS by MCs 

During the sensitization phase in CHS, MCs play promoting role by interacting with or recruiting immune cells in the skin. Upon hapten exposure, MCs release tumor necrosis factor (TNF) and promote the maturation/migration of dermal DC s to the LNs, leading to the facilitation of sensitization (23, 24). MC-derived exosome may also be involved in DC maturation (25). Furthermore, MCs facilitate sensitization by amplifying the infiltration of neutrophils into the hapten exposed skin, a key step for sensitization (26, 27), by releasing TNF and histamine (26). Recruitment of neutrophils by MCs are also important for the promotion of elicitation (23, 28). In addition, MCs present antigens to T cells by acquiring major histocompatibility complex class II (MHCII) from DCs, and can facilitate elicitation (29). However, in the elicitation phase, negative regulatory functions of MCs have also been elucidated as illustrated below.

### IL-10-Dependent Regulation of CHS

Previous studies have investigated the role of skin MCs in CHS using MC-deficient mice, such as WBB6F1-*Kit^W/Wv^* (*Kit^W/Wv^*) and C57BL/6-*Kit^W-sh/W-sh^* (*Kit^W-sh/W-sh^*) mice (Kit-dependent, constitutive MC deficiency) (9). In these mice, both facilitating and regulatory roles of MCs have been reported in CHS. For example, Norman et al. reported that CHS responses in *Kit^W/Wv^* mice are reduced when it is induced by low-concentration hapten (oxazolone) and elevated when it is induced by high-concentration hapten, suggesting that MCs are necessary for the development of CHS and to limit the excess inflammation (30). Grimbaldeston et al. also reported exacerbated oxazolone-induced CHS responses in *Kit^W/Wv^* and *Kit^W-sh/W-sh^* mice. This exacerbation was eliminated by the transfer of wild-type (WT) mouse-derived MCs, but not IL-10-deficient (IL-10^-/-^) mouse-derived MCs, suggesting that MCs limit inflammation in CHS in an IL-10-dependent manner (14). Using IL-10 reporter mice, it was later confirmed that MCs produce IL-10 most abundantly in the skin one day after the elicitation in oxazolone-induced CHS, whereas MCs produce minimal IL-10 when CHS was induced with low concentrations of oxazolone (15).

Because Kit-dependent MC-deficient mice have various hematological abnormalities other than MC deficiency, it has been debated whether the exacerbated CHS response in these mice is solely attributable to MC deficiency. However, exacerbated CHS responses have also been observed in Kit-independent MC-deficient mice (9, 11), and MC-specific depletion of IL-10 (*Mcpt5-Cre^+^Il10^f/f^* mice) recapitulated the exacerbated CHS phenotype in MC-deficient mice (15). These results indicate that MCs play a suppressive role in eliciting oxazolone-induced CHS *via* IL-10 production. In contrast, these regulatory functions of MCs were not observed by Dudeck et al., in which MC-deficient mice (both Kit-dependent and Kit-independent MC-deficient mice) were treated with 2-4-dinitrofluorobenzene-induced CHS, and showed attenuated CHS responses. Furthermore, MC-specific depletion of IL-10 did not affect the CHS response (23). Thus, MCs seem to change their roles depending on the type and concentration of haptens, although the molecular mechanisms underlying the switch of MC function in each context remain unclear. CHS protocol (e.g. interval between sensitization and elicitation, skin site of sensitization), the parameter used to measure inflammation, and the timing of analysis after the elicitation (e.g. early versus late phase) are other potential variables that would influence the functions of MCs or phenotype of MC-deficient mice in CHS. In the early phase, MCs may facilitate CHS by releasing inflammatory mediators such as TNF and histamine (23), while in the late phase, MCs may limit CHS by producing anti-inflammatory mediators such as IL-10 (15).

### Maintenance of Regulatory B cells

In addition to MCs, IL-10 is produced by several cell populations, such as regulatory T cells (Tregs) and regulatory B cells (Bregs). Both cell populations play crucial roles in the termination of the CHS response (22, 31). Kim et al. reported that MCs inhibit CHS responses by maintaining the IL-10^+^ Breg number in skin-draining LNs in an IL-5-dependent manner (16). In oxazolone-induced CHS, *Kit^W-sh/W-sh^* mice showed an augmented CHS response, which was accompanied by a reduced IL-10^+^ Breg number in the skin-draining LNs. Reconstitution with MCs restored the population of IL-10^+^ Bregs in *Kit^W-sh/W-sh^* mice and suppressed the CHS response. The level of IL-5 was markedly decreased in the LNs of *Kit^W-sh/W-sh^* mice and was restored by reconstitution of MCs. This recovery of Breg number and IL-5 level in LNs was diminished under reconstitution with IL-5^-/-^ MCs. Based on these results, it has been proposed that MCs perform regulatory functions in CHS by maintaining Breg number in LNs (16). MCs may increase IL-10 levels in peripheral tissues by producing IL-10 themselves and by maintaining IL-10-producing cell populations, such as Bregs.

### Maintenance of Resident Memory T Cells 

Skin-resident memory T (T_RM_) cells are a subset of memory T cells that provide local surveillance and do not migrate out of the skin (32). Upon inflammation, effector T cells infiltrate the skin, and some of them become T_RM_ cells, mostly CD8^+^ T_RM_ cells. IL-7, IL-15, and transforming growth factor (TGF)-β are pivotal cytokines that generate the skin T_RM_ cells (32). Skin T_RM_ cells protect the body from the invasion of pathogens; however, they are also involved in the development of various inflammatory skin diseases, including contact dermatitis.

Using oxazolone-induced CHS, Gimenez-Rivera et al. examined the relationship between MCs and CD8^+^ T_RM_ cells in skin (17). The authors modified CHS *via* repeated elicitation at long intervals (elicitation at 30 days and 60 days after the 1^st^ elicitation), which led to the accumulation of antigen-specific T_RM_ cells in the elicited skin. Under these conditions, the CHS response was significantly increased in *Kit^W-sh/W-sh^* and *Mcpt5-Cre^+^iDTR* mice. The number of CD8^+^ T_RM_ cells was significantly higher in MC-deficient mice than in control WT mice. In addition, the expression levels of IL-15, but not IL-7 and TGF-β, were upregulated in MC-deficient mice. *In vitro*, the MC proteases such as chymase and carboxypeptidase degraded IL-15. These results suggest that MCs limit skin inflammation in CHS by regulating the T_RM_ cell number *via* IL-15 degradation (17).

### Programmed Cell Death Protein-1/Programmed Cell Death-Ligand 1 Signaling

The abovementioned regulatory functions of MCs are exhibited in a contact-independent manner *via* cytokine production. However, MCs can also exhibit regulatory functions by direct contact *via* the programmed cell death-1 (PD-1, CD279)/programmed cell death-ligand 1 (PD-L1, CD274) pathway (18). The PD-1/PD-L1 pathway is a negative regulator that coordinates the balance between T cell activation and tolerance (33). PD-1 expressed on effector T cells binds to PD-L1 expressed on various tissue-resident and antigen-presenting cells and transduces signals that inhibit the proliferation of T cells and their effector functions.

To investigate the mechanisms by which the PD-1/PD-L1 pathway regulates T cell activation in CHS, we induced CHS in *Pdl1^-/-^* mice and found that *Pdl1^-/-^* and WT mice treated with the anti-PD-L1 antibody exhibited an exacerbated CHS response, indicating that the PD-1/PD-L1 pathway is important for the negative regulation of the CHS response. However, the effect of the anti-PD-L1 antibody on the CHS response was abolished in MC-deficient mice, suggesting that MCs are involved in PD-1/PD-L1-mediated negative regulation of effector T cells. Skin MCs highly express PD-L1 in both mice and humans. MCs interact with T cells in the skin, as revealed by two-photon microscopic observations. In a co-culture system of effector T cells and MCs, effector T cell activation was induced by WT MCs, which was further enhanced by *Pdl1^-/-^* MCs or *via* the blockade of the PD-1/PD-L1 pathway by the anti-PD-L1 antibody. These results suggest that MCs directly contact effector T cells in the skin and negatively regulate their activation *via* the PD-1/PD-L1 pathway (18).

## Atopic Dermatitis

Atopic dermatitis (AD) is a common chronic inflammatory skin disease. Type 2 cytokines, especially IL-4 and IL-13, are central mediators that induce various symptoms in AD, such as pruritus and skin barrier defects (34). The number of MCs is significantly increased in the skin lesions of AD, suggesting the involvement of MCs in the establishment of AD. Similar to the studies on CHS, both pro- and anti-inflammatory functions of MCs have been reported in the AD model, which may be dependent on the model used in the study.

### Regulatory Function of MCs in AD: Maintenance of Tregs (Oxazolone-Induced AD Model)

As mentioned in section 1, oxazolone is a representative hapten to induce CHS, a type 1 immune response in the skin. Single time application of oxazolone following the sensitization (at intervals of 5-7 days) induces CHS. However, repeated and frequent application of oxazolone (5-10 times at intervals of a few days) causes type 2 cytokine-shifted inflammation in the skin and is used as an AD model (35). *Kit^W-sh/W-sh^* mice exhibited exacerbated dermatitis, whereas adoptive transfer of WT MCs into *Kit^W-sh/W-sh^* mice decreased the inflammatory response, indicating that MCs play a suppressive role in this AD model (19). The suppressive effects of MC reconstitution were not observed in *Il2^-/-^* MCs, suggesting that MC-derived IL-2 is involved in the suppression mechanism. Since the ratio of activated T cells to Tregs in the skin was significantly higher in *Kit^W-sh/W-sh^* mice reconstituted with *Il2^-/-^* MCs than in those reconstituted with WT MCs, it was concluded that MCs inhibit dermatitis by maintaining Tregs *via* IL-2 production (19). Exacerbated phenotypes in *Kit^W-sh/W-sh^* mice have also been reported in the MC903-induced AD model (36), but the underlying mechanisms by which MCs suppress inflammation in this model remain unclear.

### Pro-Inflammatory Function of MCs in AD (House Dust Mite/Staphylococcal Enterotoxin B -Induced AD Model)

Repeated treatment with *Dermatophagoides farinae* (house dust mite: HDM) extract and staphylococcal enterotoxin B (SEB) induces skin inflammation that mimics AD (37). Clinical scores were significantly lower in *Kit^W-sh/W-sh^* mice and Kit-independent MC-deficient mice (*Cpa3-Cre* ^+^ *Mcl-1f/f*) than in the corresponding WT mice, indicating that MCs are required for maximal skin inflammation in this model (38). Since the Fc receptor for IgE-deficient (*FcεRI^-/-^*) mice also showed reduced skin inflammation in this model, the authors speculated that MCs facilitate atopic skin inflammation *via* FcεRI-dependent mechanisms. In another study using the same HDM/SEB-induced AD model, Serhan et al. revealed that MCs promote atopic skin inflammation by interacting with the nociceptive sensory neurons (nociceptors) (39). HDM directly activates nociceptors, which produce substance P. Substance P induces the activation of MCs surrounding nociceptors *via* the Mas-related G protein-coupled receptor B (MRGPRB), a receptor for cationic molecules from the MRGPR family. These results suggest that after exposure to HDM allergens, nociceptors sense the allergens and induce MC degranulation *via* the activation of MRGPRB on MCs, leading to the initiation of type 2 inflammation.

Therefore, in AD lesions, MCs may play facilitating roles *via* the release of various chemical mediators by degranulation, which may induce both IgE-dependent (FcεRI-mediated) and independent (MRGPRB-mediated) responses. In contrast, MCs may play regulatory roles in AD by maintaining Treg number and suppressing Th2 cell activation.

## Malignant Melanoma

Malignant melanoma (MM) is one of the most malignant types of skin cancer (40). Previous studies on the involvement of MCs in MM development have mostly been based on histological analysis investigating the number of MCs surrounding the MM and its relation to prognosis. Some reports have shown a correlation between tumor progression and the number of MCs surrounding the tumors (41, 42), whereas others have shown a decrease in the number of MCs in advanced MM (43, 44); therefore, the results have not been consistent. Recent studies using mouse MM models have shown both beneficial and detrimental roles of MCs in MM development (20, 45).

Kaesler et al. reported the beneficial role of MCs in the antitumor response in MM (45). The authors first identified that patients exhibiting better responses to anti-cytotoxic T-lymphocyte antigen 4 (CTLA4) antibody treatment developed colitis as an immune-mediated adverse effect with a systemic lipopolysaccharide (LPS) signature. In a mouse model of MM using the B16 melanoma cell line, LPS treatment reduced the tumor volume. MC-deficient mice (*Mcpt5-cre^+^* R*-DTA^fl/fl^*) showed increased tumor volume compared to control WT mice, and the suppressive effect of LPS treatment on tumor volume was not observed in MC-deficient mice. Based on these results, the authors concluded that the effective immune control of MM by the anti-CTLA4 antibody was dependent on LPS-activated MCs, which recruit tumor-infiltrating effector T cells *via* secretion of C-X-C motif chemokine ligand 10 (45).

In contrast, Somasundaram et al. reported an association between MCs and resistance to anti-PD-1 therapy in MM (20). Using humanized mice and melanomas from human patients, the authors first examined the immune cell changes in tumors after treatment with anti-PD-1 antibodies. Immunohistochemical and flow cytometric analyses revealed that MC numbers were increased in the group resistant to anti-PD-1 antibodies. Furthermore, RNA sequence analysis of tumors from patients with MM before and after anti-PD-1 antibody treatment showed an increase in MC number after treatment, especially in the non-responder group. Meanwhile, the elimination of MCs with sunitinib enhanced the therapeutic effects of the anti-PD1 antibody in the humanized MM model. Mechanistically, numerous Treg infiltrations were observed around the MCs at tumor sites, correlating with decreased MHC class I expression in the surrounding tumors. These findings suggest that MCs may promote tumor progression by downregulating tumor MHC class I expression by recruiting Tregs, either directly or indirectly. Histamine from MCs may contribute to tumor promotion by enhancing peritumoral M2 macrophage differentiation and suppressing CD8^+^ T cell activity (21). These results suggest that the depletion of MCs or downregulation of MC function can be used as potential therapeutic strategies for MM.

## Discussion

As described above, MCs perform immunosuppressive functions *via* diverse mechanisms, at least in mice (**Table 1**). At the initial stage of inflammation or when the extent of inflammation is low, MCs may play a role in promoting inflammation. However, at the late stage of inflammation or when inflammation becomes excessive, MCs may switch their role from pro- to anti-inflammatory and fine-tune inflammation to avoid undesired tissue damage (11, 15). In the cancer environment, where the activation of innate and acquired immune systems is necessary for the elimination of tumors, such regulatory functions by MCs may become deleterious to the host.

However, many open questions are unanswered. For example, signals that induce the regulatory functions of skin MCs are not yet fully understood. As for the signals that induce IL-10 production from skin MCs, a few pathways, such as vitamin D receptor signaling-dependent pathway and the IgE signaling-dependent pathway, have been reported (12, 46). Analysis of the signal-transducing molecules in those pathways may become a clue to understand the molecular mechanisms by which MCs acquire regulatory functions. Besides, skin MCs may be heterogenous populations as reported in MCs in other organs such as lung (47) and esophagus (48), and MCs with regulatory functions, such as IL-10-producing MCs, may be a particular MC subset in the skin. Indeed, while skin MCs under physiological state are maintained by local proliferation in adult skin (5, 6), bone marrow-derived MCs appear in the skin in inflammatory state (49, 50), suggesting that skin MCs may be heterogenous populations (skin resident MCs versus bone marrow-derived MCs). We are currently investigating the functional differences between those MC populations. In addition, since there are various discrepancies in the pathogenesis of human diseases and mouse disease models, elucidating the physiological significance of MCs in human diseases remains a challenge. Skin organoids/human skin equivalent systems as intermediates between mouse disease models and human patients constitute a promising strategy that will likely lead to a better understanding of the role of MC in skin diseases. Human cutaneous MCs produce minimal IL-10 (51, 52); therefore, whether IL-10-mediated regulation by MCs, as revealed by mouse studies, is actually involved in human diseases remains to be verified. Nevertheless, elucidation of the molecular mechanisms that control MC function may lead to the development of novel therapeutic strategies for inflammatory skin diseases and skin cancers.

## Author Contributions

All authors listed have made a substantial, direct, and intellectual contribution to the work and approved it for publication.

## Funding

This work was supported by the Japan Society for the Promotion of Science KAKENHI (JP19K08790, JP15H05906 [T.H.], Japan Science, Japan Agency for Medical Research and Development (AMED) (19ek0410062s0201) [T.H.], Kose Cosmetology Research Foundation [T.H.], Lydia O’leary Memorial Pias Dermatological Foundation [T.H.]

## Conflict of Interest

The authors declare that the research was conducted in the absence of any commercial or financial relationships that could be construed as a potential conflict of interest.

## Publisher’s Note

All claims expressed in this article are solely those of the authors and do not necessarily represent those of their affiliated organizations, or those of the publisher, the editors and the reviewers. Any product that may be evaluated in this article, or claim that may be made by its manufacturer, is not guaranteed or endorsed by the publisher.
